# Therapeutic efficacy of albendazole against soil-transmitted helminthiasis in children measured by five diagnostic methods

**DOI:** 10.1371/journal.pntd.0007471

**Published:** 2019-08-01

**Authors:** Johnny Vlaminck, Piet Cools, Marco Albonico, Shaali Ame, Mio Ayana, Giuseppe Cringoli, Daniel Dana, Jennifer Keiser, Maria P. Maurelli, Leonardo F. Matoso, Antonio Montresor, Zeleke Mekonnen, Greg Mirams, Rodrigo Corrêa-Oliveira, Simone A. Pinto, Laura Rinaldi, Somphou Sayasone, Eurion Thomas, Jozef Vercruysse, Jaco J. Verweij, Bruno Levecke

**Affiliations:** 1 Department of Virology, Parasitology and Immunology, Ghent University, Merelbeke, Belgium; 2 Center for Tropical Diseases, Sacro Cuore Don Calabria Hospital, Negrar, Italy; 3 Department of Life Sciences and Systems Biology, University of Turin, Turin, Italy; 4 Laboratory Division, Public Health Laboratory-Ivo de Carneri, Chake Chake, United Republic of Tanzania; 5 Jimma University Institute of Health, Jimma University, Jimma, Ethiopia; 6 Department of Veterinary Medicine and Animal Production, University of Naples Federico II, Naples, Italy; 7 Department of Medical Parasitology and Infection Biology, Swiss Tropical and Public Health Institute, Basel, Switzerland; 8 University of Basel, Basel, Switzerland; 9 Laboratory of Molecular and Cellular Immunology, Research Center René Rachou—FIOCRUZ, Belo Horizonte, Brazil; 10 Nursing school, Federal University of Minas Gerais, Brazil; 11 Department of Control of Neglected Tropical Diseases, World Health Organization, Geneva, Switzerland; 12 Techion Group Ltd, Dunedin, New Zealand; 13 Lao Tropical and Public Health Institute, Ministry of Health, Vientiane, Lao People's Democratic Republic; 14 Techion Group Ltd, Aberystwyth, United Kingdom; 15 Laboratory for Medical Microbiology and Immunology, Elisabeth-TweeSteden Hospital, Tilburg, The Netherlands; University of Cambridge, UNITED KINGDOM

## Abstract

**Background:**

Preventive chemotherapy (PC) with benzimidazole drugs is the backbone of soil-transmitted helminth (STH) control programs. Over the past decade, drug coverage has increased and with it, the possibility of developing anthelmintic resistance. It is therefore of utmost importance to monitor drug efficacy. Currently, a variety of novel diagnostic methods are available, but it remains unclear whether they can be used to monitor drug efficacy. In this study, we compared the efficacy of albendazole (ALB) measured by different diagnostic methods in a head-to-head comparison to the recommended single Kato-Katz.

**Methods:**

An ALB efficacy trial was performed in 3 different STH-endemic countries (Ethiopia, Lao PDR and Tanzania), each with a different PC-history. During these trials, stool samples were evaluated with Kato-Katz (single and duplicate), Mini-FLOTAC, FECPAK^G2^, and qPCR. The reduction rate in mean eggs per gram of stool (ERR) and mean genome equivalents / ml of DNA extract (GERR) were calculated to estimate drug efficacy.

**Principal findings and conclusions:**

The results of the efficacy trials showed that none of the evaluated diagnostic methods could provide reduction rates that were equivalent to a single Kato-Katz for all STH. However, despite differences in clinical sensitivity and egg counts, they agreed in classifying efficacy according to World Health Organization (WHO) guidelines. This demonstrates that diagnostic methods for assessing drug efficacy should be validated with their intended-use in mind and that other factors like user-friendliness and costs will likely be important factors in driving the choice of diagnostics. In addition, ALB efficacy against STH infections was lower in sites with a longer history of PC. Yet, further research is needed to identify factors that contribute to this finding and to verify whether reduced efficacy can be associated with mutations in the β-tubulin gene that have previously been linked to anthelmintic resistance.

**Trial registration:**

ClinicalTrials.gov NCT03465488.

## Author summary

During the last decade, the scale of deworming programs that aim to eliminate the morbidity caused by intestinal worms has increased to a level that is unprecedented in history. It is therefore of utmost importance to monitor any change in therapeutic efficacy that may arise from emerging drug resistance. Currently, a variety of novel methods have been described, but it remains unclear whether they can be used for monitoring drug efficacy. We applied different diagnostic methods to measure the efficacy of a commonly administered drug in deworming programs in 3 countries with different historical exposure to deworming programs. Compared to the standard diagnostic method, all diagnostic methods revealed good agreement in classifying the therapeutic efficacy according to World Health Organization guidelines, despite clear differences in diagnostic performance. We also noticed that the drug efficacy was lower in countries where drug pressure has been high. However, more research is necessary to identify factors that explain this variation in drug efficacy, including but not limited to the frequency in mutations in genes that are known to be linked with anthelmintic resistance.

## Introduction

Infections with soil-transmitted helminths (STHs; *Ascaris lumbricoides*, *Trichuris trichiura*, *Necator americanus* and *Ancylostoma duodenale*) are responsible for the highest burden among all neglected tropical diseases. Recent global estimates indicate that in 2015, more than 1.6 billion people were infected with at least one of the four STH species [1], resulting in a global burden of approximately 1.9 million disability-adjusted life years [2]. Preventive chemotherapy (PC) or the periodical administration of a single-oral dose of albendazole (ALB; 400 mg) or mebendazole (MEB; 500 mg) to preschool- (PSAC) and school-aged children (SAC) is the main strategy to control the morbidity caused by STHs [3]. In 2017, global coverage of PC in at-risk populations was nearly 70%, though the target is to reach 75% coverage by 2020, and to eventually eliminate soil-transmitted helminthiasis as a public health problem [4–6]. The latter target is defined by reaching less than 1% moderate and heavy intensity infections in SAC [5].

The downside of these increased control efforts is that resistance to anthelmintic drugs, such as ALB and MEB, is likely to develop. Both drugs belong to the same drug class (benzimidazole (BZ) drugs) and share the same mode of action. Moreover, they are administered in single doses that usually do not achieve 100% efficacy [7–10]. Should anthelmintic resistance (AR) against these BZ drugs eventually emerge and spread, it will jeopardize PC-based control of STH due to the few acceptable alternative treatment options [11, 12]. All this reinforces the urgent need to promote accessibility of anthelmintic drugs with different modes of action, alone or in combination, and a thoroughly designed surveillance system that detects any changes in anthelmintic drug efficacy arising through the evolution of AR.

The World Health Organization advises to monitor drug efficacy in case treatment failure is suspected or–regardless of suspected drug failure–when drugs have been administered in PC-programs for at least four years [13]. To monitor the efficacy of anthelmintic drugs against STHs, WHO currently recommends measuring the reduction in number of STH eggs excreted in stool after drug administration (egg reduction rate, ERR) using either a single Kato-Katz thick smear or the McMaster method [13].

Recently, novel methods have been introduced in the field of STH diagnostics, including Mini-FLOTAC [14, 15], FECPAK^G2^ [16, 17] and the DNA-based diagnostic methods such as quantitative PCR (qPCR) [18–20]. Each of these methods offers one or more advantages over the recommended methods, pertaining to increased clinical sensitivity [15, 18, 21–23] and specificity (qPCR is able to differentiate different helminths at the species level) [24–27], quality assurance (FECPAK^G2^ automatically stores images of each sample which can be consulted at any time [17, 28]; qPCR includes internal controls within each run [29]), flexibility as to when samples are examined (for both Mini-FLOTAC and qPCR, stool can be preserved for analysis at a later time point [23, 25, 30–32]). Although each of these novel methods has recently been used to evaluate drug efficacy [16, 33, 34], there remains a paucity of studies that perform a head-to-head comparison of the drug efficacy obtained by different diagnostic methods. Moreover, these studies tested the hypothesis that the methods provide significantly different ERR estimates. Rather, the correct hypothesis is to assess whether these differences are within the bounds of equivalence. As illustrated in supplementary information (**S1 Info**), the absence of a significant difference does not imply equivalent ERR estimates nor does the presence of a significant difference rule out equivalent ERR results.

Therefore, in this study we compared the equivalence in ALB efficacy measured by duplicate Kato-Katz thick smear, Mini-FLOTAC, FECPAK^G2^ and qPCR in a head-to-head comparison with a single Kato-Katz thick smear. For this, a drug efficacy trial with ALB was performed in three different countries (Ethiopia, Lao PDR and Pemba (Tanzania)) with different historical levels of drug exposure.

## Methods

### Ethics statement

The study protocol has been reviewed and approved by the Institutional Review Board (IRB) of the Faculty of Medicine and Health Sciences of Ghent University, Belgium (Ref. No B670201627755; 2016/0266). The trial protocol was subsequently reviewed and approved by the IRBs associated with each trial site (Ethical Review Board of Jimma University, Jimma, Ethiopia: RPGC/547/2016; National Ethics Committee for Health Research, Vientiane, Lao PDR: 018/NECHR; Zanzibar Medical Research and Ethics Committee, United Republic of Tanzania: ZAMREC/0002/February/2015 and the IRB from Centro de Pesquisas René Rachou, Belo Horizonte, Brazil: 2.037.205). The trial was retrospectively registered on Clinicaltrials.gov (ID: NCT03465488) on March 7, 2018.

Parent(s)/guardians of participants signed an informed consent document indicating that they understood the purpose and procedures of the study, and that they allowed their child to participate. If the child was ≥5 years, he or she had to orally assent in order to participate. Participants of ≥12 years of age were only included if they signed an informed consent document indicating that they understood the purpose and the procedures of the study, and were willing to participate.

### Study design and population

The selection of the study sites was based on their experience in assessing drug efficacy, evaluating the performance of diagnostic methods, the availability of well-equipped diagnostic facilities and skilled personnel, and PC-history [35]. Based on the reported national coverage of drug administration to both PSAC and SAC for the last 5 years (2009–2014; Preventive Chemotherapy Database of the WHO), the site in Ethiopia was considered to have experienced a low drug exposure, the site in Lao PDR a medium drug exposure and the site in Pemba (Tanzania) a high drug exposure prior to the start of the trials [35] (**Table 1**). Note that the initial study protocol included a study site in Brazil. However, due to the low number of cases on which not all diagnostic methods were performed, the site was excluded from this report.

**Table 1:** Location and treatment history of the four study sites.

Country	District/Province/State	Treatment history
**Brazil**	Minas Gerais	PC since 2007 (ALB)
**Ethiopia**	Jimma	PC since 2015 (ALB)
**Lao PDR**	Nam Bak	PC since 2007 (MEB)
**Tanzania**	Pemba Island	PC since 1994 (ALB)

PC: preventive chemotherapy, ALB: albendazole, MBZ: mebendazole.

The trials were designed to assess an equivalence in treatment efficacy of a single oral dose of 400 mg ALB against STH infections in SAC measured by a variety of diagnostic methods. The study focused on SAC (age 5–14) since they are the major target of PC programs, and they usually represent the group with highest worm burdens for *A*. *lumbricoides* and *T*. *trichiura* [36]. Subjects were not included in the study if they could not provide a stool sample at baseline or follow-up and had active diarrhea or any other acute medical condition at baseline. Children with a known hypersensitivity to ALB or MEB, who received anthelmintic treatment within 90 days prior to the start of the trial were and did not swallow the entire drug tablet or vomited within four hours following drug ingestion were also excluded from the study.

At the start of each trial, schools were visited by the local principal investigator and a team of field officers, who explained the planned trial and sampling method to the parents and teachers and the children. At baseline, SAC were asked to provide a fresh stool sample, after which they were administered a single oral dose of 400 mg ALB under supervision. The ALB used in the different studies originated from the same production batch (GlaxoSmithKline Batch N°: 335726) and was provided by WHO. All collected stool samples were kept in a cooler with ice packs while transported to the laboratory, where they were processed on the same day of collection. Stool samples were processed to determine the fecal egg counts (FECs; expressed in eggs per gram of stool (EPG)) for each STH using Kato-Katz (single and duplicate), Mini-FLOTAC and FECPAK^G2^. As FECs of the latter technique could not finalized on the day of sample collection (see section Diagnostic methods), results of the FECPAK^G2^ technique were not used to select individuals for inclusion at follow-up. Aliquots of a subset of the baseline samples were preserved in ethanol for molecular analysis. Preliminary data has indicated that downstream analysis of STH β-tubulin genes was very challenging when egg concentration was low (data not published). Therefore, only samples with a FEC of ≥150 EPG for at least one STH species were withheld for further molecular analysis. Fourteen to 21 days after drug administration, a second stool sample was collected from all the children that were found positive for any STH by duplicate Kato-Katz or Mini-FLOTAC at baseline. Stool samples collected at follow-up were again examined by Kato-Katz (single and duplicate), Mini-FLOTAC and FECPAK^G2^. Aliquots from all follow-up samples were preserved for further molecular analysis regardless of the FECs.

### Sample size calculation

A sample size was calculated to test the hypothesis that FECPAK^G2^, Mini-FLOTAC and duplicate Kato-Katz provide equivalent drug efficacy results measured by ERRs compared to a single Kato-Katz. This sample size calculation did not include the qPCR method. Given the differences in drug efficacy of ALB across the STH species [8, 9] (*A*. *lumbricoides*: ~99%, hookworms: ~96%, *T*. *trichiura*: ~65%), a level of equivalence that is acceptable for *T*. *trichiura* may not be acceptable for *A*. *lumbricoides*. We therefore applied a level of equivalence that was tailored to the different STH species. The level of equivalence for *A*. *lumbricoides*, hookworms and *T*. *trichiura* was set arbitrarily at -/+2.5, -/+5.0 and -/+10-point percentage respectively. This means that a method provides equivalent drug efficacy estimates as single Kato-Katz if the confidence intervals surrounding the mean difference in drug efficacy does not exceed these set of values (**S1 Info**). To calculate the corresponding sample size for each of the STH species, a simulation study was performed that considered (i) the variation in ERR and baseline FECs both across and within STH species, (ii) the variation in FECs introduced by the egg counting process, (iii) the paired ERR results across diagnostic methods, and (iv) a post-hoc correction for a pair-wise comparison. Based on this simulation, at least 110, 100 and 12 complete cases are required for *T*. *trichiura*, hookworm and *A*. *lumbricoides*, respectively. A detailed description of the sample size calculation is available elsewhere [35].

### Diagnostic methods

Upon arrival in the laboratory, stool samples were homogenized with a wooden spatula and subsequently subjected to microscopic examination by means of single and duplicate Kato-Katz, Mini-FLOTAC and FECPAK^G2^. Two aliquots of 0.5 g stool were also preserved in an Eppendorf tube containing 1 ml of absolute ethanol for later DNA extraction and qPCR analysis. Detailed standard operating procedures (SOPs) for the different diagnostic methods were published earlier [35, 37]. Here we briefly mention the most important steps for each of the methods.

For Kato-Katz, two slides were prepared (slide A and B) and examined for the presence of STH eggs within 30–60 min following preparation. The results of slide A represented the results of a single Kato-Katz and egg counts were multiplied by 24 to obtain the FECs (expressed as EPG). The sum of the egg counts obtained after reading slide A and B represented the results for duplicate Kato-Katz and were multiplied by 12 to obtain the FECs.

For Mini-FLOTAC, we homogenized 2 g of fresh stool with 38 ml of flotation solution (saturated salt solution, density = 1.20) in the Fill-FLOTAC recipient [15]. After transferring the suspension into the two chambers of the Mini-FLOTAC device, the device was placed on a horizontal surface for 10 min after which the reading disk was translated. Finally, both Mini-FLOTAC chambers were screened for the presence of STH eggs. The number of eggs counted were multiplied by 10 to obtain the FECs.

The FECPAK^G2^ method was performed as described by Ayana et al. [17]. Briefly, stool was homogenized in tap water in a Fill-FLOTAC device [15], after which it was transferred into a FECPAK^G2^ sedimenter to allow STH eggs to sediment. The following day, the supernatant was poured off and saturated saline solution (specific density = 1.2) was added to the remaining slurry. The whole content of the sedimenter was then poured into a FECPAK^G2^ filtration unit from which 2 separate aliquots were taken and transferred to 2 wells of a FECPAK^G2^ cassette. Following an accumulation step of at least 20 minutes, the cassettes were placed in the Micro-I device for image capture. The device automatically imaged both wells and stored the images prior to uploading them to the FECPAK^G2^ server. Finally, the mark-up technician identified and counted any STH eggs present in the images using specialized software. Mark-up of the images was not performed on the day of examination and hence the results were not used to select individuals for inclusion in follow-up. Results of the mark-up were saved automatically for reporting and analysis. For FECPAK^G2^ the eggs counted in both wells were multiplied by 34 to calculate the FECs.

For quality control purposes, a predefined, randomly selected subset of samples (10% of the total number of samples) was re-evaluated by each of the three egg count methods. To this end, a senior researcher, who was blinded to the initial FECs, re-counted STH eggs across all three egg count methods. A third examiner would re-count STH eggs in case of discrepancies. An in-depth analysis of these quality control results will be published in a separate manuscript.

In order to perform qPCR, DNA was extracted from the preserved stool samples and analyzed for the presence of DNA of STH at the Laboratory for Medical Microbiology and Immunology (Elisabeth-TweeSteden Hospital, Tilburg, The Netherlands) as part of two multiplex qPCR assays [35]. The variation between runs was monitored by means of the Cq values of the positive controls (DNA template for each STH species). We defined the variation between runs negligible when the difference in Cq values of the positive controls between runs did not exceed 1. The inhibition of the qPCR assay was controlled by adding a known quantity of phocine herpes virus DNA in each DNA extract and by subsequently quantifying this virus’ DNA by qPCR. Inhibition was present in the sample when the difference in Cq-value between the virus’ DNA in a clinical sample and a pure virus DNA sample did not exceed 1. We did not observe a difference in Cq across controls of more than 1 Cq, nor did we observe inhibition of the qPCRs in any of the samples (quality control results will be published in a follow-up manuscript). For each target species, qPCR results were expressed as genomic equivalents per ml of DNA extract (GE/ml). The reported qPCR results for hookworms were calculated as the sum of GE/ml of both hookworm species (*Ancylostoma* and *Necator americanus*).

### Statistical analysis

A sample was considered positive for a STH infection if it tested positive on at least one diagnostic method (duplicate Kato-Katz, Mini-FLOTAC, FECPAK^G2^ or qPCR). The efficacy of a single oral dose of 400 mg ALB is reported separately for each STH species and for each microscopic method by means of ERR, using the following formula: ERR (%) = 100% x [1- (arithmetic mean FEC at follow-up / arithmetic mean FEC at baseline)]. For qPCR, a similar formula was used, where FEC was replaced by DNA concentration (GE/ml), yielding the genome equivalent reduction rate (GERR): GERR (%) = 100% x [1- (arithmetic mean DNA concentration at follow-up / arithmetic mean DNA concentration at baseline)]. A bootstrap analysis was used to determine the corresponding 95% confidence intervals (95%CI) around the (G)ERR point estimate for each diagnostic method and the difference in drug efficacy compared to a single Kato-Katz across diagnostic methods. A permutation test was used to assess the equivalence in (G)ERR between single Kato-Katz thick smear and either duplicate Kato-Katz, Mini-FLOTAC, FECPAK^G2^ or qPCR. Bonferroni’s correction was applied for multiple comparison between methods (level of significance was set at 0.0125 = 0.05 / 4 comparisons).

The (G)ERR point estimates were used to classify drug efficacy as ‘satisfactory’, ‘doubtful’ or ‘reduced’ following the WHO criteria recommended for a single Kato-Katz [13] (**Table 2**). In addition, the agreement between a single Kato-Katz and the other methods in the assignment of drug efficacy into ‘satisfactory’, ‘doubtful’ and ‘reduced’ was evaluated by Fleiss’ kappa statistic (κ_Fleiss_). The value of this statistic indicates a slight (κ_Fleiss_ <0.2), fair (0.2≤ κ_Fleiss_ <0.4), moderate (0.4≤ κ_Fleiss_ <0.6), substantial (0.6≤ κ_Fleiss_, <0.8) or an almost perfect agreement (κ_Fleiss_ ≥0.8).

**Table 2:** Classification of the efficacy of a single oral dose 400 mg albendazole against soil-transmitted helminths. The efficacy is measured as the reduction in mean fecal egg counts following drug administration (ERR). These thresholds were recommended by the World Health Organization for a single Kato-Katz.

Helminth species	Satisfactory	Doubtful	Reduced
***Ascaris lumbricoides***	≥95%	85%≤ ERR <95%	<85%
***Trichuris trichiura***	≥50%	40%≤ ERR <50%	<40%
**Hookworm**	≥90%	80%≤ ERR <90%	<80%

Finally, we also assessed the distribution of individual responses measured across the five diagnostic methods. Individual ERR (iERR) were calculated using the following formula: iERR = 100% x [1- (FEC at follow-up of individual *i* / FEC at baseline of individual *i*)]. Individual GERR (iGERR) were calculated using the following formula: iGERR = 100% x [1- (DNA concentration at follow-up of individual *i* / DNA concentration at baseline of individual *i*)]. We classified the individual response for each STH species and for each method into ‘cured’ (no eggs/DNA was found in follow-up sample), ‘satisfactory’, ‘doubtful’ or ‘reduced’ (see Table 1) and ‘absent’ (drug efficacy was below zero due to higher egg counts or DNA-concentration in the follow-up sample than in baseline sample). Subsequently, the agreement between a single Kato-Katz and the other methods in the assignment of individual drug efficacy was assessed by Fleiss’ kappa statistic (κ_Fleiss_). All statistical analyses were performed in R [38]. Graphs were produced using R.

## Results

### Demographics and STH status of the complete cases

**Fig 1:**
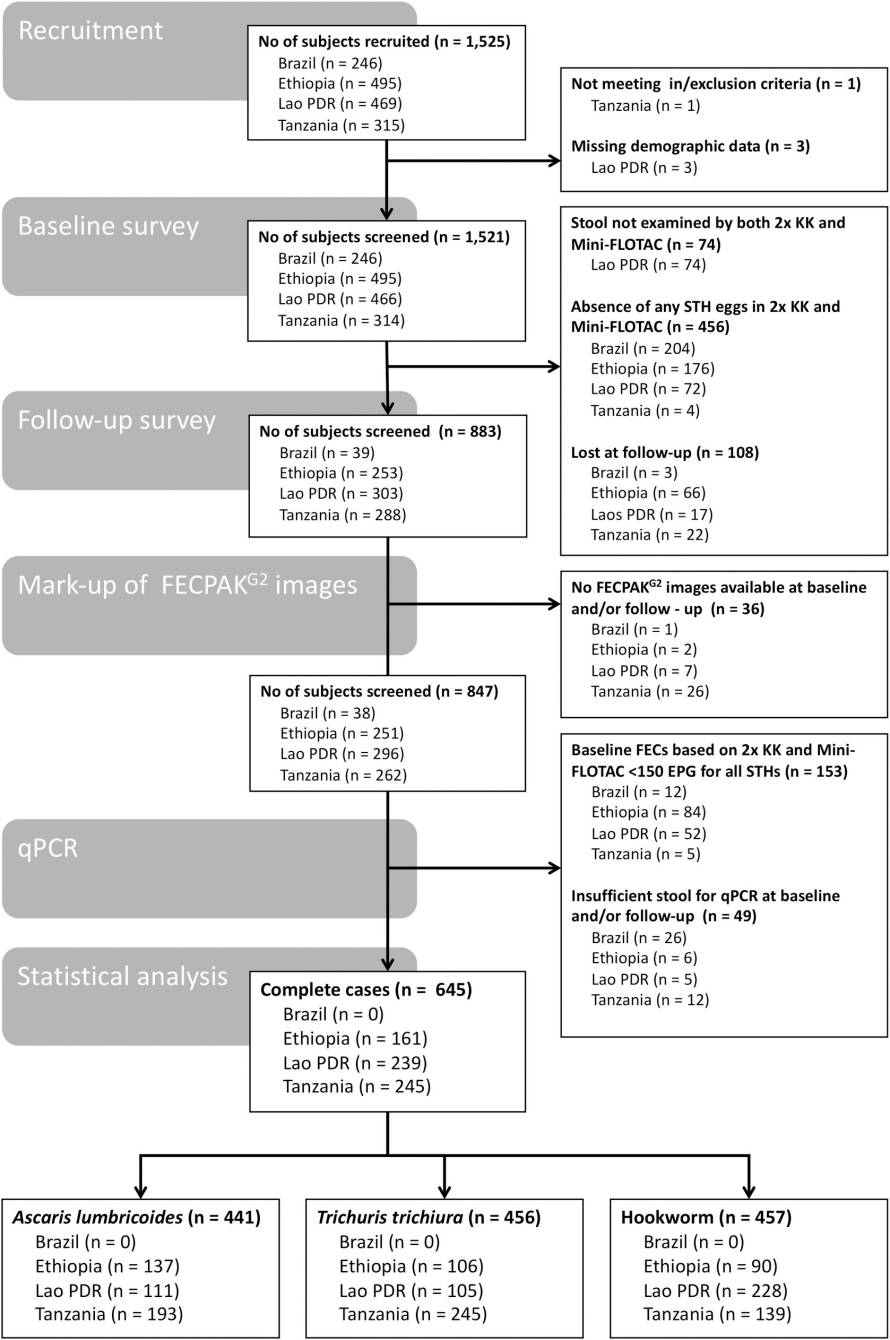
Number of subjects withheld at recruitment, baseline and follow-up, and for statistical data analysis. FECs: fecal egg counts expressed in eggs per gram of stool (EPG), 2x KK: duplicate Kato-Katz.

The number of children that were withheld after recruitment and at baseline or follow-up visits, and those that were eventually incorporated in the final statistical analysis are summarized in **Fig 1**. Complete data was available for 645 children across three of the four study sites (Ethiopia: 161 cases; Lao PDR: 239 cases; Pemba (Tanzania): 245 cases).

With the exception of Pemba (Tanzania), where more females (n = 137) were included than males (n = 108), the sex ratio (males:females) was approximately 1:1 in all study sites. The median age (25^th^ and 75^th^ quantile) of the children across the three study sites equaled 11.0 years (9.0; 12.0). The participants in Ethiopia (9.0 years [8.0; 10.0]) were slightly younger than those in Lao PDR (12.0 years [11.0; 13.0]) and Pemba (Tanzania; 11.0 years [10.0; 12.0]). In total, there were 441 complete cases for *A*. *lumbricoides* (Ethiopia: 137, Lao PDR: 111, Pemba (Tanzania): 193), 456 for *T*. *trichiura* (Ethiopia: 106, Lao PDR: 105, Pemba (Tanzania): 245) and 457 for hookworm (Ethiopia: 90, Lao PDR: 228, Pemba (Tanzania): 139). The qPCR results showed that all individuals who excreted hookworm eggs were infected with *N*. *americanus*. Both *N*. *americanus* and *Ancylostoma* DNA was detected in only 8 individuals from one school in Pemba (Tanzania). The qPCR results for hookworm used in our calculations represent the combined GE/ml detected for both species. Due to the nature of the school selection procedure (prioritization of schools where STH prevalence was expected to be moderate to high and premature discontinuation of recruitment in a school when the prevalence of STH was low), the number of complete cases is not equally distributed across the schools. A minority of the schools actually provide the majority of the infected children (**S2 Info**).

### Equivalence in therapeutic drug efficacy

**Tables 3–5** describe the efficacy of ALB measured by the different diagnostic methods across the three study sites for *A*. *lumbricoides*, *T*. *trichiura* and hookworm, respectively. For *A*. *lumbricoides* infections, efficacy of a single-oral dose of 400 mg ALB estimated by single Kato-Katz was high (ERR >95%) across the different study sites (**Table 3**). This high drug efficacy was confirmed by the three microscopic methods as well as by qPCR. The absolute point percent difference in drug efficacy did not exceed 2% (duplicate Kato-Katz: 0–0.1%; Mini-FLOTAC: 0.1%– 0.6%; FECPAK^G2^: 0.0%– 1.8%; qPCR: 0.0%– 0.8%). All diagnostic methods provided significantly equivalent estimates of drug efficacy compared to a single Kato-Katz (i.e. the 95%CI around the difference in drug efficacy between diagnostic methods did not include 2.5%), except for FECPAK^G2^ in Pemba (Tanzania) and qPCR in both Ethiopia and Pemba (Tanzania), where evidence of equivalent drug efficacy was marginal since CIs included the 2.5% bounds of equivalence (FECPAK^G2^: [-5.7%; 0.8%]; qPCR: [-6.5%; 5.5%] in Pemba (Tanzania) and [-0.1%; 4.3%] in Ethiopia).

**Table 3:** Equivalence of ALB drug efficacy against *Ascaris lumbricoides* measured by five diagnostic methods. Mean intensity of infection corresponds with the mean fecal egg counts (FEC; expressed as eggs per gram of stool (EPG)) for single (1x KK) and duplicate (2x KK) Kato-Katz, Mini-FLOTAC and FECPAK^G2^ and with the mean DNA concentration (expressed as genome equivalents per ml of DNA (GE/ml)) for qPCR. For *A*. *lumbricoides*, there is significant evidence that a diagnostic method results in equivalent drug efficacy results compared to a 1x KK when the 95% confidence intervals (95%CI) around the difference in drug efficacy does not include +/-2.5%. Significant evidence of equal drug efficacy results is indicated by ‘*’. For *A*. *lumbricoides*, drug efficacy was classified as ‘satisfactory’ when drug efficacy ≥95%, reduced when drug efficacy was less than 85% or doubtful in all other cases.

*A*. *lumbricoides*		Mean intensity of infection at baseline	Mean intensity of infection at follow-up	Drug efficacy (%) [95%CI]	Difference in drug efficacy compared to 1x KK [95%CI]	Drug efficacy based on WHO thresholds
**Ethiopia (n = 137)**						
	1x KK	7,869.5	6.0	99.9 [99.8; 100]	-	Satisfactory
	2x KK	7,937.6	5.6	99.9 [99.8; 100]	0.0 [0.0; 0.0]*	Satisfactory
	Mini-FLOTAC	4,408.4	7.4	99.8 [99.6; 100]	0.1 [0.0; 0.3]*	Satisfactory
	FECPAK^G2^	1,622.3	2.7	99.8 [99.5; 100]	0.1 [-0.1; 0.4]*	Satisfactory
	qPCR	15,466.0	145.8	99.1 [96.7; 100]	0.8 [-0.1; 4.3]	Satisfactory
**Lao PDR (n = 111)**						
	1x KK	13,028.5	105.9	99.2 [97.7; 100]	-	Satisfactory
	2x KK	13,045.2	104.6	99.2 [97.8; 99.9]	0.0 [-0.1; 0.0]*	Satisfactory
	Mini-FLOTAC	5,592.9	66.4	98.8 [97.5; 100]	0.6 [-0.2; 1.6]*	Satisfactory
	FECPAK^G2^	2,711.1	23.0	99.2 [97.0; 99.9]	0.0 [-1.0; 1.2]*	Satisfactory
	qPCR	16,210.5	150.6	99.1 [97.6; 99.8]	0.1 [-1.2; 1.5]*	Satisfactory
**Pemba (Tanzania) (n = 193)**						
	1x KK	14,371.8	458.5	96.8 [92.1; 99.5]	-	Satisfactory
	2x KK	15,075.0	504.2	96.7 [91.5; 99.6]	0.1 [-0.2; 1.0]*	Satisfactory
	Mini-FLOTAC	11,135.5	369.0	96.7 [96.5; 99.8]	0.1 [-1.4; 1.4]*	Satisfactory
	FECPAK^G2^	6,322.1	90.0	98.6 [92.5; 99.4]	-1.8 [-5.7; 0.8]	Satisfactory
	qPCR	54,790.6	1,735.9	96.8 [93.1; 99.3]	0.0 [-6.5; 5.5]	Satisfactory

**Table 4:** Equivalence of drug efficacy of albendazole against *Trichuris trichiura* measured by five diagnostic methods. Mean intensity of infection corresponds with the mean fecal egg counts (FEC; expressed as eggs per gram of stool (EPG)) for single (1x KK) and duplicate (2x KK) Kato-Katz, Mini-FLOTAC and FECPAK^G2^ and with the mean DNA concentration (expressed as genome equivalents per ml of DNA (GE/ml)) for qPCR. For *T*. *trichiura*, there is significant evidence that a diagnostic method results in equivalent drug efficacy results compared to a 1x KK when the 95% confidence intervals (95%CI) around the difference in drug efficacy does not include +/-10%. Significant evidence of equal drug efficacy results is indicated by ‘*’. For *T*. *trichiura*, drug efficacy was classified as ‘satisfactory’ when drug efficacy ≥50%, reduced when drug efficacy was less than 40% or doubtful in all other cases.

*T*. *trichiura*		Mean intensity of infection at baseline	Mean intensity of infection at follow-up	Drug efficacy (%) [95%CI]	Difference in drug efficacy compared to 1x KK [95%CI]	Drug efficacy based on WHO thresholds
**Ethiopia (n = 106)**						
	1x KK	206.7	97.4	52.9 [39.1; 65.7]	-	Satisfactory
	2x KK	195.2	101.3	48.1 [33.2; 62.7]	4.8 [-0.6; 10.4]	Doubtful
	Mini-FLOTAC	147.0	93.3	36.5 [14.9; 56.2]	16.4 [-1.0; 36.4]	Reduced
	FECPAK^G2^	15.1	12.2	19.1 [-27.8; 55.8]	33.8 [-8.5; 101.0]	Reduced
	qPCR	770.4	250.5	67.5 [27.5; 83.8]	14.6 [-39.5; 39.3]	Satisfactory
**Lao PDR (n = 105)**						
	1x KK	356.8	225.8	36.7 [-0.6; 57.8]	-	Reduced
	2x KK	359.1	213.7	40.5 [4.4; 61.0]	3.8 [-12.0; 3.1]	Doubtful
	Mini-FLOTAC	228.5	119.0	47.9 [23.9; 64.3]	11.2 [-49.8; 13.9]	Doubtful
	FECPAK^G2^	48.6	41.4	14.7 [-54.1; 47.0]	22.0 [-41.2; 126.4]	Reduced
	qPCR	628.7	656.5	-4.4 [-78.9; 41.7]	41.1 [-32.7; 152.6]	Reduced
**Pemba (Tanzania) (n = 245)**						
	1x KK	3,110.5	3,459.1	-11.2 [-30.4; 5.9]	-	Reduced
	2x KK	3,384.0	3,549.2	-4.9 [-23.8; 12.0]	6.3 [-14.9; 1.9]	Reduced
	Mini-FLOTAC	3,463.5	2,949.3	14.8 [-0.6; 28.8]	26.0 [-45.2; -9.2]	Reduced
	FECPAK^G2^	495.6	488.9	1.3 [-24.7; 21.3]	12.5 [-43.2; 21.7]	Reduced
	qPCR	12,657.9	6,461.5	49.0 [4.5; 73.3]	60.2 [-99.8; -2.0]	Doubtful

**Table 5:** Equivalence of drug efficacy of albendazole against hookworm infections measured by five diagnostic methods. Mean intensity of infection corresponds with the mean fecal egg counts (FEC; expressed as eggs per gram of stool (EPG)) for a single (1x KK) and duplicate (2x KK) Kato-Katz, Mini-FLOTAC and FECPAK^G2^ and with the mean DNA concentration (expressed as genome equivalents per ml of DNA (GE/ml)) for qPCR. For hookworm, there is significant evidence that a diagnostic method results in equivalent drug efficacy results compared to a 1x KK when the 95% confidence intervals (95%CI) around the difference in drug efficacy does not include +/- 5%. Significant evidence of equal drug efficacy results is indicated by ‘*’. For hookworm, drug efficacy was classified as ‘satisfactory’ when drug efficacy ≥90%, reduced when drug efficacy was less than 80% or doubtful in all other cases.

Hookworm		Mean intensity of infection at baseline	Mean intensity of infection at follow-up	Drug efficacy (%) [95%CI]	Difference of drug efficacy compared to 2x KK [95%CI]	Drug efficacy based on WHO thresholds
**Ethiopia (n = 90)**						
	1x KK	247.7	9.1	96.3 [92.8; 98.5]	-	Satisfactory
	2x KK	242.1	9.1	96.3 [92.7; 98.3]	0.0 [-0.5; 0.9]*	Satisfactory
	Mini-FLOTAC	153.2	9.8	93.6 [87.9; 97.1]	2.7 [-2.1; 9.0]	Satisfactory
	FECPAK^G2^	103.5	0.8	99.3 [97.3; 100]	3.0 [-8.2; 0.9]	Satisfactory
	qPCR	20,653.4	1,150.1	94.4 [88.8; 97.7]	1.9 [-2.4; 7.6]	Satisfactory
**Lao PDR (n = 228)**						
	1x KK	2,079.1	81.8	96.1 [93.7; 97.7]	-	Satisfactory
	2x KK	2,043.8	76.9	96.2 [94.0; 97.8]	-0.1 [-0.6; 0.2]*	Satisfactory
	Mini-FLOTAC	820.0	46.7	94.3 [90.6; 96.8]	1.8 [-0.6; 6.0]	Satisfactory
	FECPAK^G2^	657.8	17.0	97.4 [95.6; 98.7]	-1.3 [-3.7; 0.9]*	Satisfactory
	qPCR	273,720.7	14,827.6	94.6 [89.2; 97.6]	1.5 [-2.1; 7.7]	Satisfactory
**Pemba (Tanzania) (n = 139)**						
	1x KK	285.4	45.2	84.2 [75.4; 90.7]	-	Doubtful
	2x KK	286.9	47.1	83.6 [74.4; 90.3]	0.6 [-1.5; 3.2]*	Doubtful
	Mini-FLOTAC	215.5	32.0	85.1 [74.7; 91.8]	-0.9 [-11.8; 9.0]	Doubtful
	FECPAK^G2^	141.9	13.7	90.3 [81.4; 95.7]	-6.1 [-15.9; 3.0]	Satisfactory
	qPCR	127,139.3	25,941.6	79.6 [58.7; 92.6]	4.6 [-10.8; 31.4]	Reduced

For *T*. *trichiura* infections, ALB efficacy estimations varied significantly depending on the study site and the diagnostic method that was used (**Table 4**). Estimations obtained by single Kato-Katz were 52.9% in Ethiopia, 36.7% in Lao PDR and -11.2% in Pemba (Tanzania). There was a large deviation from the drug efficacy measured by single Kato-Katz and those based on the other diagnostic methods. The absolute point percent difference between single Kato-Katz and the other methods was the smallest for duplicate Kato-Katz (4.8%– 6.3%) and the largest for qPCR (14.6%– 60.2%). None of the methods provided equal drug efficacy results (CI around the difference in drug efficacy between diagnostic methods included the 10% bounds of equivalence). Moreover, clear difference could be noted across the different methods. In contrast to the other methods, drug efficacy measured by a duplicate Kato-Katz was marginally equivalent to those obtained by single Kato-Katz with the CI just including the 10-point percent (Ethiopia: [-0.6%; 10.4%]; Lao PDR: [-12.0%; 3.1%] and Pemba (Tanzania): [-14.9%; 1.9%]).

For hookworm infections (**Table 5**), the drug efficacy measured by single Kato-Katz was high (>95%) in both Ethiopia (96.3%) and Lao PDR (96.1%), but moderate in Pemba (Tanzania) (84.2%). Overall, these drug efficacy estimates were confirmed by the other diagnostic methods. The absolute point percent differences in drug efficacy did not exceed 7% (duplicate Kato-Katz: 0%– 0.6%; Mini-FLOTAC: 0.9%– 2.7%; FECPAK^G2^: 1.3%– 6.1%; qPCR: 1.5%– 4.6%). A duplicate Kato-Katz provided equivalent drug efficacy estimates across all study sites (the CI around the difference in drug efficacy between diagnostic methods did not include the 5% bounds of equivalence, Ethiopia: [95%CI: -0.5; 0.9], Lao PDR: [95%CI: -0.6; 0.2]); Pemba (Tanzania) [95%CI: -1.5; 3.2]). The only other significant equivalent drug efficacy result was found for FECPAK^G2^ in Lao PDR (95%CI: -3.7; 0.9). The remaining pair-wise comparisons in both Ethiopia (Mini-FLOTAC: [95%CI: -2.1; 9.0]; FECPAK^G2^: [95%CI: -8.1; 0.9]; qPCR: [95%CI: -2.4; 7.6]) and Lao PDR (Mini-FLOTAC: [95%CI: -0.6; 6.0%]; qPCR: [95%CI: -2.1; 7.7]) indicated that drug efficacies were marginally equivalent, the 95%CI just including the 5% equivalence threshold. This was in contrast to the findings from Pemba (Tanzania) where no clear evidence of equivalent drug efficacies for Mini-FLOTAC [95%CI: -11.8; 9.0%], FECPAK^G2^ [95%CI: -15.9; 3.0] and qPCR [95%CI: -10.8; 31.4] was observed.

In summary, a duplicate Kato-Katz provided drug efficacy significantly or marginally equivalent to a single Kato-Katz for the three STH species in all study sites. For the other methods, the equivalence of drug efficacy varied by STH species and study site.

### Agreement in classifying drug efficacy

**Table 6** summarizes the agreement in classifying the efficacy of ALB between single Kato-Katz and the other diagnostic methods across the three study sites for each STH species. For both duplicate Kato-Katz (κ_Fleiss_ = 0.81, *p* <0.001) and qPCR (κ_Fleiss_ = 0.84, *p* <0.001) there was almost a perfect agreement (κ_Fleiss_ ≥0.8). Both these methods agreed with a single Kato-Katz in 7 out of the 9 observations. For FECPAK^G2^ (κ_Fleiss_ = 0.65, *p* = 0.01) there was a substantial agreement (0.61 ≤ κ_Fleiss_ <0.81), for MiniFLOTAC (κ_Fleiss_ = 0.60, *p* = 0.03) there was a moderate agreement (0.41 ≤ κ_Fleiss_ <0.61).

**Table 6:** Agreement in classifying the efficacy of albendazole between single Kato-Katz and duplicate Kato-Katz, Mini-FLOTAC, FECPAK^G2^ and qPCR. The cross tables represent the agreement in classifying the efficacy of albendazole as ‘satisfactory’, ‘doubtful’ or ‘reduced’, between a single Kato-Katz (1x KK) and a duplicate Kato-Katz (2x KK), Mini-FLOTAC, FECPAK^G2^ and qPCR across the three study sites and for each soil-transmitted helminth species (9 observations). Drug efficacy is based on egg reduction rate (ERR) for microscopic methods and by means of reduction in genome equivalents (GERR) for qPCR. World Health Organization criteria to define drug efficacy into ‘satisfactory’, ‘doubtful’ and ‘reduced’ were previously recommended for a single Kato Katz (see **Table 2**).

		1x KK			κ_Fleiss_, *p*-value
		Satisfactory	Doubtful	Reduced	
2x KK	Satisfactory	5	0	0	0.81, p <0.001
	Doubtful	1	1	1	
	Reduced	0	0	1	
		Satisfactory	Doubtful	Reduced	
Mini-FLOTAC	Satisfactory	5	0	0	0.60, *p* = 0.03
	Doubtful	0	1	1	
	Reduced	1	0	1	
		Satisfactory	Doubtful	Reduced	
FECPAK^G2^	Satisfactory	5	1	0	0.65, *p* = 0.01
	Doubtful	0	0	0	
	Reduced	1	0	2	
		Satisfactory	Doubtful	Reduced	
qPCR	Satisfactory	6	0	0	0.84, *p* <0.001
	Doubtful	0	0	1	
	Reduced	0	1	1	

### Individual drug responses

**Fig 2:**
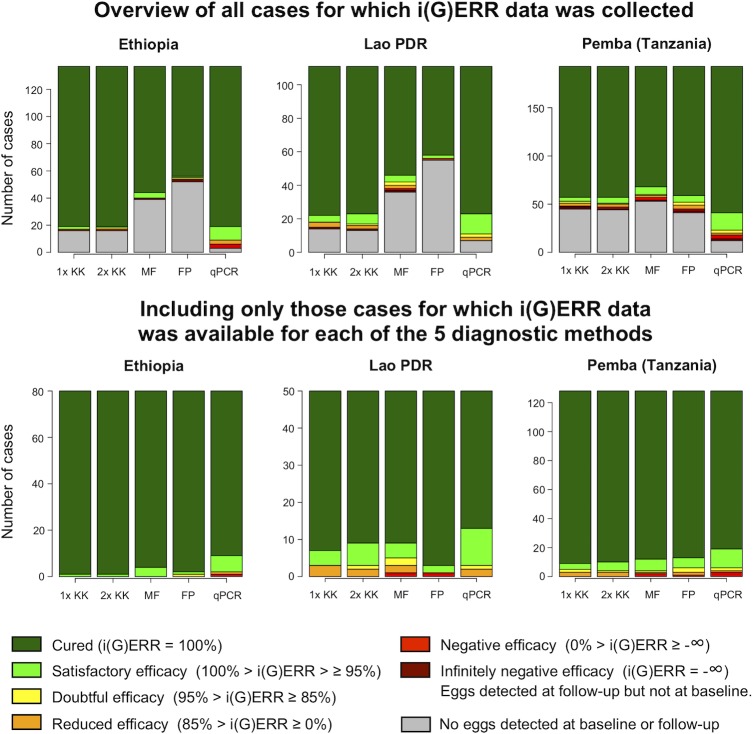
The individual drug efficacy of albendazole against *Ascaris lumbricoides*, measured by five diagnostic methods. The upper three bar plots illustrate the different classifications of individual albendazole efficacy for *A*. *lumbricoides* detected in the study population (n = 441; Ethiopia: 137, Lao PDR: 111, Pemba (Tanzania): 193) in each of the 3 countries using 5 different diagnostic methods (single (1x KK) and duplicate Kato-Katz (2x KK), Mini-FLOTAC (MF), FECPAK^G2^ (FP) and qPCR). The bars represent the number of cases. The color of the bars reflects the individual egg or genome equivalent reduction rate (i(G)ERR). The bottom three bar plots represent the classification of the i(G)ERRs for *A*. *lumbricoides* for those individuals for which i(G)ERR data was available for all five diagnostic methods (n = 258; Ethiopia: 80, Lao PDR: 50, Pemba (Tanzania): 128).

**Fig 3:**
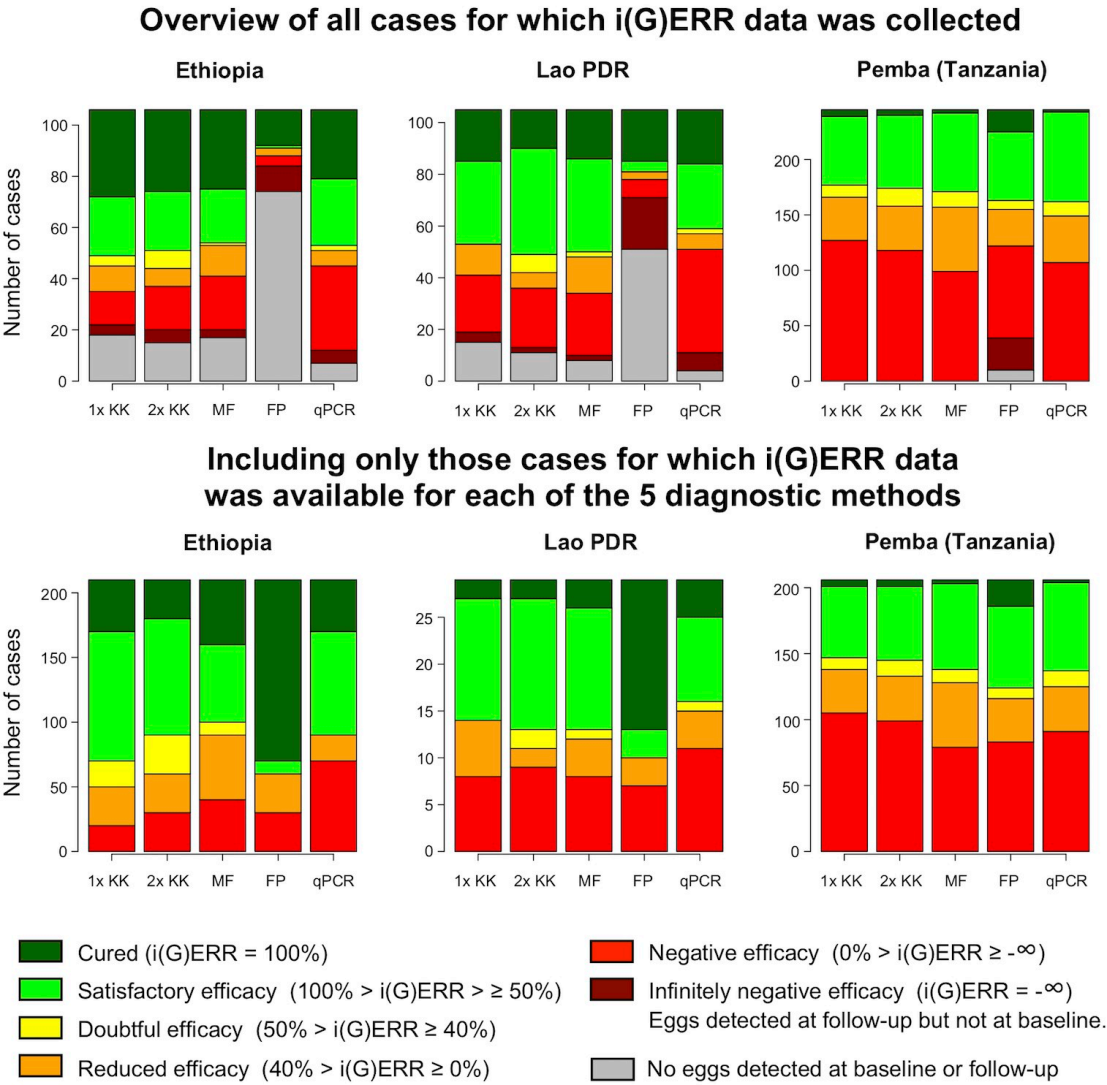
The individual drug efficacy of albendazole against *Trichuris trichiura*, measured by five diagnostic methods. The upper three bar plots illustrate the different classifications of individual albendazole efficacy for *T*. *trichiura* detected in the study population (n = 456; Ethiopia: 106, Lao PDR: 105, Pemba (Tanzania): 245) in each of the 3 countries using 5 different diagnostic methods (single (1x KK) and duplicate Kato-Katz (2x KK), Mini-FLOTAC (MF), FECPAK^G2^ (FP) and qPCR). The bars represent the number of cases. The color of the bars reflects the individual egg or genome equivalent reduction rate (i(G)ERR). The bottom three bar plots represent the classification of the i(G)ERRs for *T*. *trichiura* for those individuals for which i(G)ERR data was available for all five diagnostic methods (n = 256; Ethiopia: 21, Lao PDR: 29, Pemba (Tanzania): 206).

**Fig 4:**
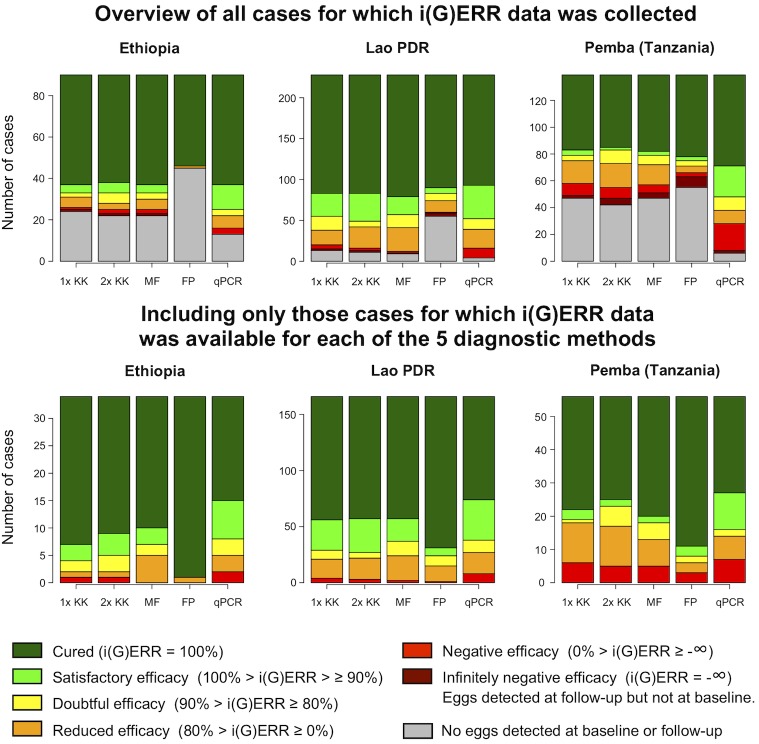
The individual drug efficacy of albendazole against hookworm, measured by five diagnostic methods. The upper three bar plots illustrate the different classifications of individual ALB efficacy for hookworm detected in the study population (n = 457; Ethiopia: 90, Lao PDR: 228, Pemba, Tanzania: 139) in each of the 3 countries using 5 different diagnostic methods (single (1x KK) and duplicate Kato-Katz (2x KK), Mini-FLOTAC (MF), FECPAK^G2^ (FP) and qPCR). The bars represent the number of cases. The color of the bars reflects the individual egg or genome equivalent reduction rate (i(G)ERR). The bottom three bar plots represent the classification of the i(G)ERRs for hookworm for those individuals for which i(G)ERR data was available for all five diagnostic methods (n = 256; Ethiopia: 34, Lao PDR: 166, Pemba, Tanzania: 56).

**Figs 2–4** provide an overview of individual drug responses to ALB for the five diagnostic methods across the different sites for *A*. *lumbricoides*, *T*. *trichiura* and hookworm, respectively. The top three bar plots illustrate the different classifications of individual drug efficacy for that helminth species detected in the study population in each of the three countries by five different diagnostic methods. Based on their i(G)ERR, individuals are classified into one of seven different categories, which in turn correspond with a specific color, ranging from dark green (completely cured, i(G)ERR = 100%) to dark red (eggs/DNA detected at follow-up but not at baseline, i(G)ERR = -∞). The grey part of the bar represents the individuals for whom no efficacy could be calculated since no eggs or DNA was detected for that species at baseline and follow-up. The bottom three bar plots represent the individual drug efficacy for those 770 individuals for whom i(G)ERR was measured with each of the five diagnostic methods.

Generally, these figures highlight three important findings. First, they confirm the distinct differences in sensitivity across the diagnostic methods. FECPAK^G2^ was previously evaluated as being less sensitive than Kato-Katz, while qPCR was found to have superior sensitivity for all STH [16, 26, 39]. This is also supported by the results of our study, where we noticed high numbers of false negative test results at baseline and follow-up for FECPAK^G2^. When applying FECPAK^G2^, drug efficacy could not be measured in 438 (32.3%) of the 1,354 individuals with STH infection, because of false negative results at baseline (*A*. *lumbricoides*: 148/441, *T*. *trichiura*: 135/456 and hookworms: 155/457). In contrast, when applying qPCR, individual drug efficacy could not be measured in only 56 (4.1%) of the 1,354 individuals because of false negative results at baseline (top graphs of **Figs 2–4**: *A*. *lumbricoides*: 22/441, *T*. *trichiura*: 11/456 and hookworm: 23/456).

Second, they indicate that there are a number of cases where eggs or DNA were found at follow-up, but not at baseline (this mathematically results in an infinite increase of eggs or DNA at follow-up or an individual drug efficacy of minus infinity (dark red portion of bars in upper panels). Overall, these types of cases were observed by at least one diagnostic method in 8% of the total number of cases (n = 1,354), but differences across diagnostic methods and STH were observed. They were more prevalent when the FECPAK^G2^ method was used (5.5% = 75/1,354). For the other diagnostic methods, the proportion of samples that were positive at follow-up but negative at baseline did not exceed 1.3% (single Kato-Katz: 1.2%; duplicate Kato-Katz: 1.3%; Mini-FLOTAC: 1.0%; qPCR: 1.2%), the majority being *T*. *trichiura* cases (17.2% = 78/456). The cases were less frequently observed for hookworm (5.3% = 24/457) and *A*. *lumbricoides* (1.8% = 8/441).

Third, they indicate that variation in individual drug response across STH and countries is similar across diagnostic methods. This is most obvious when we focus on the cases for which an individual drug efficacy response was available for all methods (bottom panels of **Figs 2–4**). When employing a single Kato-Katz, the highest drug efficacy was observed for *A*. *lumbricoides* followed by hookworms and *T*. *trichiura*. For *A*. *lumbricoides*, 96.9% (= 250/258) of the individuals showed a drug response that was at least satisfactory (light green + dark green). For hookworm, this proportion equaled 79.7% (= 204/256), whereas for *T*. *trichiura* this was only 34.4% (= 88/256).

For *A*. *lumbricoides*, the proportions of individuals with at least satisfactory drug efficacy (light green + dark green) as measured by single Kato-Katz were comparable across the 3 countries (Ethiopia: 100% (= 80/80); Lao PDR: 94.0% (= 47/50) and Pemba (Tanzania): 96.1% (= 123/128)) (bottom graphs of **Figs 2–4**). For *T*. *trichiura*, fewer individuals showed satisfactory drug efficacy to ALB in Pemba (Tanzania) compared to Lao PDR or Ethiopia (Pemba (Tanzania): 28.6% (= 59/206) *vs*. Lao PDR: 51.7% (= 15/29) or Ethiopia: 66.7% (= 14/21)). When investigating the individual ALB response to hookworm, fewer individuals show satisfactory drug efficacy in Pemba (Tanzania) (66.1% (= 37/56)) compared to Lao PDR (82.5% (= 137/166)) or Ethiopia (88.2% (= 30/34)). Regardless of the diagnostic method used, the same trends in individual efficacy were apparent across STH species and countries.

Cross tables were made to gain more insights into the agreement between individual drug response across the different methods (**S**3 **Info**). These tables illustrate the agreement of calculated i(G)ERR using single Kato-Katz and the four other methods for *A*. *lumbricoides*, *T*. *trichiura* and hookworms, respectively. For a duplicate Kato-Katz an almost perfect agreement (κ_Fleiss_ ≥ 0.80) was observed for each of the STH (*A*. *lumbricoides*: 0.96 *T*. *trichiura*: 0.91; hookworm: 0.96, *p* <0.001). For Mini-FLOTAC, there was an almost perfect agreement for *A*. *lumbricoides* (κ_Fleiss_ = 0.88, p <0.001) and a substantial agreement for the other two STH (*T*. *trichiura*: 0.62; hookworm: 0.79, *p* <0.001). For FECPAK^G2^, there was moderate agreement for *A*. *lumbricoides* (κ_Fleiss_ = 0.55, *p* <0.001) and a fair agreement for the remaining STH (*T*. *trichiura*: 0.31; hookworm: 0.39, *p* <0.001). For qPCR, there was a substantial agreement for hookworms (κ_Fleiss_ = 0.61, *p*< 0.001), moderate agreement for both *A*. *lumbricoides* (κ_Fleiss_ = 0.59, *p* <0.001), and a fair agreement for *T*. *trichiura* (κ_Fleiss_ = 0.36, *p* <0.001).

## Discussion

The present study evaluated the efficacy of ALB against STH infections in three different endemic study sites using five different diagnostic methods. The rationale for this study was twofold. First, we wanted to evaluate if the different diagnostic methods provide equivalent drug efficacy results compared to a single Kato-Katz (the WHO recommended method) and to ultimately make recommendations on which diagnostic methods can be used for assessing drug efficacy. The second goal was to evaluate the ALB efficacy against STH in all three study sites with varying anthelmintic drug pressure histories. The presented study is unique in a number of ways. It is the first study that performs a multi-country, standardized, head-to-head comparison of established (single and duplicate Kato-Katz) and novel microscopic (Mini-FLOTAC and FECPAK^G2^) and molecular (qPCR) diagnostic methods for assessing drug efficacy against STHs. This study was not designed to prove that ERR estimates differ across methods, rather it verified whether methods are equivalent in assessing drug efficacy, which, as illustrated in **S1 Info**, is a subtle, but important difference.

### No single diagnostic method provides ERR that are equivalent to single Kato-Katz for all STH, but they agree in classifying drug efficacy according to the WHO guidelines

We found that none of the evaluated tests provided equivalent results to those obtained by single Kato-Katz for all three STH. However, this conclusion needs to be interpreted with some caution. First, the species-specific levels of equivalence (the predefined bounds of equivalence) are arbitrary and likely to be set too strict. For instance, setting the level of equivalence at 10% for *T*. *trichiura* might be too strict for Pemba (Tanzania) given that ALB efficacy measured by duplicate Kato-Katz was -11.2%. On the other hand, the sample size was initially determined to compare the microscopic methods only (See [35]). By adding the qPCR results to this comparison, we increased the number of comparisons from 3 to 4. Consequentially, the level at which significant equivalence could be shown was reduced (0.05/4 = 0.0125 instead of 0.05/3 = 0.0166). Moreover, the sample size calculation was performed bases on certain assumptions regarding the ERR and FECs across and within STH species, which might have resulted in an underestimation of the true variation in the population [40, 41]. However, despite the lack of equivalence, for most methods there was relatively good agreement in classifying ALB efficacy according to WHO guidelines. This suggests that each method holds promise for the assessment of drug efficacy in the context of assessing drug efficacy within STH control programs.

### Diagnostic methods for assessing drug efficacy need to be validated for their intended-use

The results of the present study highlight that the impact of the diagnostic sensitivity on ERR results is minimal (**Tables 3–5** and **Figs 2–4**). Although there were substantial differences in FECs across the different microscopic methods, this did not have a major impact on the equivalence of ERR. For example, the FECs for *A*. *lumbricoides* based on single Kato-Katz were at least double of those based on FECPAK^G2^ across all study sites (Ethiopia: 7,870 EPG *vs*. 1,622 EPG; Lao PDR: 13,029 EPG *vs*. 2,711 EPG; Pemba (Tanzania): 14,372 EPG *vs*. 6,322 EPG), yet at each study site both methods agreed that the efficacy is still satisfactory. This agreement in drug efficacy, despite the clear differences in diagnostic sensitivity and FECs, are in line with previous studies involving both animal [42] and human helminths [43–45], and underscore that diagnostic methods need to be to be validated for their intended-use. Moreover, it highlights that other aspects such as user-friendliness and operational costs might become pivotal factors when deciding to recommend or use any given method. Additionally, it should be noted that our findings for qPCR do not necessarily apply for other qPCR assays, given that the plethora of described qPCR assays for STHs can differ substantially in performance. It is also important to point out that these findings are based on results obtained in sites where STH prevalence and intensities of STH infections are still relatively high. It is possible that the impact of the diagnostic sensitivity of a method on ERR calculations, as illustrated in animals, will increase when working in settings with very low infection intensities [46, 47]

### The efficacy of single ALB against was lower in sites where drug pressure has been high

We strategically selected the different study sites to cover a wide range of drug pressure. In our study, the study site in Ethiopia was least exposed to BZ drugs, followed by the one in Lao PDR. On Pemba (Tanzania), BZ drugs had been most frequently administered. When focusing on the drug efficacy estimated by single Kato-Katz, there was an obvious trend between the drug pressure and drug efficacy for each of the three STH species. The ERRs dropped as a function of historic drug pressure. The declining trend was most pronounced for *T*. *trichiura*, for which ERR ranged from 52.9% in Ethiopia over 36.7% in Lao PDR to -11.2% in Pemba (Tanzania). For both *A*. *lumbricoides* and hookworm, the efficacy of ALB was highest in both Ethiopia and Lao PDR (*A*. *lumbricoides*: ~ 99% and hookworms: ~96%), and lowest on Pemba, Tanzania (*A*. *lumbricoides*: 96.8%; hookworms: 84.2%). Whether this reduced drug efficacy on Pemba (Tanzania) is indicative for the emergence of AR remains to be verified since it has been shown that other factors may contribute to a reduced efficacy. For example, it has been described that the efficacy of ALB against *T*. *trichiura* infections declines as a function of increasing infection intensity [48]. This also seems to be the case in the present study, where we notice a trend between average infection intensity (Pemba (Tanzania): 3,111 EPG; Lao PDR: 357 EPG; Ethiopia: 207 EPG) and reduced drug efficacy (Pemba (Tanzania): -11.2% ERR; Lao PDR: 36.7% ERR; Ethiopia: 52.9% ERR by single Kato-Katz). It is also possible that both these processes occur simultaneously and mutually enhance the noticed effects of reduced drug efficacy. Poor drug efficacy could result in increasing transmission and more subjects being infected with a large number of worms.

To further assess the emergence of AR, we will analyze the frequency of known single nucleotide polymorphisms (SNPs) in the β-tubulin gene at codons 167 (TTC to TAC), 198 (GAA to GCA) and 200 (TTC to TAC) in a subset of the baseline and follow-up samples [35]. Subsequently, individual-based drug efficacy models will be built to explore the association between the frequency of these SNPs and other factors, including, but not limited to, infection intensity [49]. The results of this analysis will be presented and discussed in detail in a follow-up paper. At present, only a few studies with small sample sizes originating from a limited number of endemic areas have been performed to assess the association between *β*-tubulin SNPs and reduced anthelmintic efficacy in human STH [33, 50–55]. In these studies, it was noted that polymorphisms were predominantly found in codon 200 of the *β*-tubulin gene and that these mutations were more abundant in a *T*. *trichiura* worm population following drug administration. Nevertheless, no association could ever be proven with reduced drug efficacy in any STH species. Overall, there are limited reports of declining or poor drug efficacy [9, 33, 56, 57]. Of note, some of these studies were flawed in terms of their design or analysis. For example, Krücken and colleagues reported a poor efficacy of ALB against *A*. *lumbricoides* infections in Rwandan SAC, but the study findings might have been negatively affected by the fact that follow-up sampling occurred too soon after drug administration (7–10 days), which likely led to the detection of eggs from dying or degenerating worms [58].

### Differential susceptibility of hookworm species to ALB

Interestingly, Pemba was the only site where both hookworm species were detected by qPCR. In eight children, mixed infections with *Ancylostoma* spp. and *N*. *americanus* were identified, confirming the finding by Albonico et al. [59]. Follow-up samples of these eight individuals were all negative for *Ancylostoma* spp. (cure rate (CR) of 100%), while two still excreted *Necator* DNA (CR = 75%). Although this was observed in only eight cases, it supports the findings on the efficacy of ALB to different helminth infections presented by Horton [60] who reported a notably lower CR for *Necator* infections (CR = 75%, 30 studies) compared to *Ancylostoma* spp. infections (CR = 92%, 23 studies). Given the seemingly differential susceptibility of both hookworm genera to ALB, it is important to differentiate hookworm infections in order to have correct efficacy estimates for each species. This is of particular interest when the possible contribution of zoonotic *A*. *ceylanicum* infections from animal reservoirs to the observed drug efficacy is investigated.

### Conclusion

The present study investigated the equivalence of five different diagnostic tools for the evaluation of anthelmintic efficacy. None of the evaluated tests provided equivalent results to those obtained by the currently recommended single Kato-Katz for all STH, but this might be due to the number of pairwise-comparisons and the strict bounds of equivalence. Overall, there was an acceptable agreement in classifying the efficacy of ALB, suggesting that each of the investigated methods holds promise to assess drug efficacy in the context of STH control programs. The results also highlight that the clinical sensitivity or the ability to accurately estimate egg counts should not be the only parameters to determine the best diagnostic tool to assess drug efficacy. Instead, there are a number of other aspects that should also be considered to make a well-founded decision on what method(s) to recommend for monitoring drug efficacy in STH control programs, like user-friendliness and operational costs per test. We observed a decreasing trend in drug efficacy as a function of increasing historic drug pressure, yet further research is needed to identify factors that are contributing to this variation and to determine whether reduced efficacy can be linked with the known β-tubulin SNPs.

## Supporting information

S1 Info Testing for difference *vs*. testing for equivalence. (DOCX)

S2 Info Number of complete cases per school, sex and age across the four study sites. (DOCX)

S3 Info Agreement in classifying the individual response against *A. lumbricoides, T. trichiura* and hookworm between a single Kato-Katz and duplicate Kato-Katz, Mini-FLOTAC, FECPAK^G2^ and qPCR. (DOCX)

S4 Info Complete dataset. (XLSX)
