# Supplementary material for: Therapeutic efficacy of albendazole against soil-transmitted helminthiasis in children measured by five diagnostic methods
Source: PLoS Negl Trop Dis. 2019 Aug 1;13(8):e0007471. doi: 10.1371/journal.pntd.0007471 (PMC6675043; doi:10.1371/journal.pntd.0007471)
Supplement: S1 Info — (DOCX) [file pntd.0007471.s001.docx]

**Supplementary Info SI1: Testing for difference vs. testing for equivalence**

In this study, we want to verify whether methods provide equivalent drug efficacy estimates measured by reduction in egg counts following drug administration (ERR). In other words, are the difference in ERR between bounds of equivalence. As a consequence of this, it is inappropriate to test for any difference in ERRs, and this is illustrated by 5 toy scenarios in the Figure below.


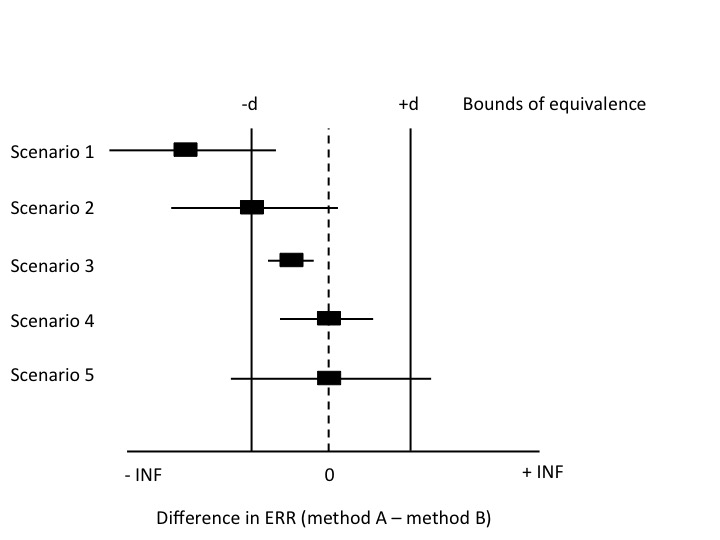


In this Figure, the x-axis represents the point percent difference in ERR between the methods A and B. The values range from minus infinity (method B providing higher ERRs), over zero (no difference in ERRs across methods) to plus infinity (method B providing lower ERRs). The straight vertical lines represent the bounds of equivalence (-d; +d) and the dashed vertical line represents the a zero difference in ERR. Methods result in significant different ERRs when the 95% confidence intervals (horizontal lines) do not include zero. This is the case for scenarios 1 and 3. Methods provide equivalent ERR results when the 95% confidence intervals do not include the bounds of equivalence, which is the case for scenario 3 and 4. These toy examples illustrate that

absence of a significant difference does not imply that methods are equivalent (scenarios 2 and 5). Moreover, they highlight that presence of a significant difference does not rule out equivalent ERR across methods (scenario 3).
