# Supplementary material for: Therapeutic efficacy of albendazole against soil-transmitted helminthiasis in children measured by five diagnostic methods
Source: PLoS Negl Trop Dis. 2019 Aug 1;13(8):e0007471. doi: 10.1371/journal.pntd.0007471 (PMC6675043; doi:10.1371/journal.pntd.0007471)
Supplement: S2 Info — (DOCX) [file pntd.0007471.s002.docx]

**Supplementary Information SI02: Number of complete cases per school, sex and age across the three study sites**

|  |  | Ethiopia  (n = 161) | Laos  (n = 239) | | Tanzania  (n = 245) |
| --- | --- | --- | --- | --- | --- |
| School ID | |  |  |  | |
|  | 01 | 143 | 13 | | 64 |
|  | 02 | 18 | 33 | | 21 |
|  | 03 |  | 151 | | 85 |
|  | 04 |  | 35 | | 75 |
|  | 05 |  | 7 | |  |
|  |  |  |  | |  |
| Sex | |  |  | |  |
|  | Female | 83 | 115 | | 137 |
|  | Male | 78 | 124 | | 108 |
|  |  |  |  | |  |
| Age (in years) | | | | | |
|  | 6 | 6 | 10 | | 0 |
|  | 7 | 20 | 11 | | 20 |
|  | 8 | 35 | 12 | | 23 |
|  | 9 | 31 | 13 | | 14 |
|  | 10 | 33 | 8 | | 28 |
|  | 11 | 13 | 23 | | 82 |
|  | 12 | 8 | 47 | | 52 |
|  | 13 | 14 | 62 | | 19 |
|  | 14 | 1 | 53 | | 7 |
