# Supplementary material for: Therapeutic efficacy of albendazole against soil-transmitted helminthiasis in children measured by five diagnostic methods
Source: PLoS Negl Trop Dis. 2019 Aug 1;13(8):e0007471. doi: 10.1371/journal.pntd.0007471 (PMC6675043; doi:10.1371/journal.pntd.0007471)
Supplement: S3 Info — (DOCX) [file pntd.0007471.s003.docx]

**Supplementary Information SI03**

**Agreement in classifying the individual response against *Ascaris* between a single Kato-Katz and a duplicate Kato-Katz, Mini-FLOTAC, FECPAK^G2^ and qPCR.** The cross tables represent the agreement in classifying the individual drug responses (= 100% x [1- (fecal egg count or DNA-load at follow-up / fecal egg count or DNA-load at baseline)]) as ‘absent’, ‘reduced’, ‘doubtful’, ‘satisfactory’ and ‘cured’, between single Kato-Katz (1x KK) and duplicate Kato-Katz (2x KK), Mini-FLOTAC (MF), FECPAK^G2^ (FP) and qPCR across 258 *Ascaris* cases for whom an i(G)ERR could be measured. The i(G)ERR was classified as ‘absent’ when the i(G)ERR < 0 (egg counts or DNA load at follow-up exceeds those measured at baseline); as ‘reduced’ when 85% > i(G)ERR ≥ 0; as ‘doubtful’ when 95% > i(G)ERR ≥ 85; as ‘satisfactory’ when 100% > i(G)ERR ≥ 95% and as ‘cured’ when i(G)ERR = 100%.

|  |  | 1x KK | | | | |
| --- | --- | --- | --- | --- | --- | --- |
|  |  | Absent | Reduced | Doubtful | Satisfactory | Cured |
| 2x KK | Absent | 0 | 0 | 0 | 0 | 0 |
|  | Reduced | 0 | 5 | 0 | 0 | 0 |
|  | Doubtful | 0 | 1 | 1 | 0 | 0 |
|  | Satisfactory | 0 | 0 | 1 | 9 | 3 |
|  | Cure | 0 | 0 | 0 | 0 | 238 |
|  |  |  |  |  |  |  |
|  |  | Absent | Reduced | Doubtful | Satisfactory | Cured |
| Mini-FLOTAC | Absent | 0 | 3 | 0 | 0 | 0 |
|  | Reduced | 0 | 3 | 0 | 0 | 0 |
|  | Doubtful | 0 | 0 | 1 | 1 | 1 |
|  | Satisfactory | 0 | 0 | 1 | 6 | 9 |
|  | Cure | 0 | 0 | 0 | 2 | 231 |
|  |  |  |  |  |  |  |
|  |  | Absent | Reduced | Doubtful | Satisfactory | Cured |
| FECPAK^G2^ | Absent | 0 | 2 | 0 | 0 | 0 |
|  | Reduced | 0 | 1 | 0 | 0 | 1 |
|  | Doubtful | 0 | 0 | 1 | 0 | 3 |
|  | Satisfactory | 0 | 0 | 0 | 5 | 5 |
|  | Cure | 0 | 3 | 1 | 4 | 232 |
|  |  |  |  |  |  |  |
|  |  | Absent | Reduced | Doubtful | Satisfactory | Cured |
| qPCR | Absent | 0 | 1 | 1 | 1 | 1 |
|  | Reduced | 0 | 2 | 0 | 1 | 1 |
|  | Doubtful | 0 | 2 | 0 | 1 | 0 |
|  | Satisfactory | 0 | 1 | 0 | 4 | 25 |
|  | Cure | 0 | 0 | 1 | 2 | 214 |

**Agreement in classifying the individual response against *Trichuris* between a single Kato-Katz and a duplicate Kato-Katz, Mini-FLOTAC, FECPAK^G2^ and qPCR.** The cross tables represent the agreement in classifying the individual drug responses (= 100% x [1- (fecal egg count or DNA-load at follow-up / fecal egg count or DNA-load at baseline)]) as ‘absent’, ‘reduced’, ‘doubtful’, ‘satisfactory’ and ‘cured’, between single Kato-Katz (1x KK) and duplicate Kato-Katz (2x KK), Mini-FLOTAC (MF), FECPAK^G2^ (FP) and qPCR across 256 *Trichuris* cases for whom an i(G)ERR could be measured. The i(G)ERR was classified as ‘absent’ when the i(G)ERR < 0 (egg counts or DNA load at follow-up exceeds those measured at baseline); as ‘reduced’ when 40% > i(G)ERR ≥ 0; as ‘doubtful’ when 50% > i(G)ERR ≥ 40; as ‘satisfactory’ when 100% > i(G)ERR ≥ 50% and as ‘cured’ when i(G)ERR = 100%.

|  |  | 1x KK | | | | |
| --- | --- | --- | --- | --- | --- | --- |
|  |  | Absent | Reduced | Doubtful | Satisfactory | Cured |
| 2x KK | Absent | 100 | 11 | 0 | 0 | 0 |
|  | Reduced | 14 | 20 | 1 | 4 | 0 |
|  | Doubtful | 0 | 5 | 9 | 3 | 0 |
|  | Satisfactory | 1 | 6 | 1 | 70 | 1 |
|  | Cure | 0 | 0 | 0 | 0 | 10 |
|  |  |  |  |  |  |  |
|  |  | Absent | Reduced | Doubtful | Satisfactory | Cured |
| Mini-FLOTAC | Absent | 66 | 15 | 3 | 7 | 0 |
|  | Reduced | 31 | 12 | 4 | 11 | 0 |
|  | Doubtful | 5 | 0 | 1 | 6 | 0 |
|  | Satisfactory | 13 | 15 | 3 | 50 | 3 |
|  | Cure | 0 | 0 | 0 | 3 | 8 |
|  |  |  |  |  |  |  |
|  |  | Absent | Reduced | Doubtful | Satisfactory | Cured |
| FECPAK^G2^ | Absent | 55 | 15 | 3 | 19 | 1 |
|  | Reduced | 20 | 5 | 5 | 9 | 0 |
|  | Doubtful | 4 | 2 | 0 | 2 | 0 |
|  | Satisfactory | 26 | 10 | 3 | 26 | 1 |
|  | Cure | 10 | 10 | 0 | 21 | 9 |
|  |  |  |  |  |  |  |
|  |  | Absent | Reduced | Doubtful | Satisfactory | Cured |
| qPCR | Absent | 59 | 24 | 6 | 20 | 0 |
|  | Reduced | 21 | 5 | 1 | 13 | 0 |
|  | Doubtful | 7 | 2 | 0 | 4 | 0 |
|  | Satisfactory | 27 | 10 | 4 | 39 | 4 |
|  | Cure | 1 | 1 | 0 | 1 | 7 |

**Agreement in classifying the individual response against hookworm between a single Kato-Katz and a duplicate Kato-Katz, Mini-FLOTAC, FECPAK^G2^ and qPCR.** The cross tables represent the agreement in classifying the individual drug responses (= 100% x [1- (fecal egg count or DNA-load at follow-up / fecal egg count or DNA-load at baseline)]) as ‘absent’, ‘reduced’, ‘doubtful’, ‘satisfactory’ and ‘cured’, between single Kato-Katz (1x KK) and duplicate Kato-Katz (2x KK), Mini-FLOTAC (MF), FECPAK^G2^ (FP) and qPCR across 256 hookworm cases for whom an i(G)ERR could be measured. The i(G)ERR was classified as ‘absent’ when the i(G)ERR < 0 (egg counts or DNA load at follow-up exceeds those measured at baseline); as ‘reduced’ when 80% > i(G)ERR ≥ 0; as ‘doubtful’ when 90% > i(G)ERR ≥ 80; as ‘satisfactory’ when 100% > i(G)ERR ≥ 90% and as ‘cured’ when i(G)ERR = 100%.

|  |  | 1x KK | | | | |
| --- | --- | --- | --- | --- | --- | --- |
|  |  | Absent | Reduced | Doubtful | Satisfactory | Cured |
| 2x KK | Absent | 9 | 0 | 0 | 0 | 0 |
|  | Reduced | 2 | 27 | 3 | 0 | 0 |
|  | Doubtful | 0 | 3 | 4 | 5 | 2 |
|  | Satisfactory | 0 | 0 | 4 | 28 | 4 |
|  | Cure | 0 | 0 | 0 | 0 | 165 |
|  |  |  |  |  |  |  |
|  |  | Absent | Reduced | Doubtful | Satisfactory | Cured |
| Mini-FLOTAC | Absent | 4 | 2 | 0 | 0 | 1 |
|  | Reduced | 5 | 17 | 7 | 3 | 3 |
|  | Doubtful | 1 | 6 | 1 | 10 | 2 |
|  | Satisfactory | 0 | 3 | 2 | 15 | 5 |
|  | Cure | 1 | 2 | 1 | 5 | 160 |
|  |  |  |  |  |  |  |
|  |  | Absent | Reduced | Doubtful | Satisfactory | Cured |
| FECPAK^G2^ | Absent | 2 | 2 | 0 | 0 | 0 |
|  | Reduced | 2 | 5 | 2 | 4 | 5 |
|  | Doubtful | 0 | 4 | 1 | 5 | 1 |
|  | Satisfactory | 0 | 3 | 0 | 5 | 2 |
|  | Cure | 7 | 16 | 8 | 19 | 163 |
|  |  |  |  |  |  |  |
|  |  | Absent | Reduced | Doubtful | Satisfactory | Cured |
| qPCR | Absent | 4 | 4 | 3 | 2 | 4 |
|  | Reduced | 4 | 13 | 1 | 8 | 3 |
|  | Doubtful | 0 | 5 | 2 | 6 | 3 |
|  | Satisfactory | 1 | 4 | 4 | 15 | 30 |
|  | Cure | 2 | 4 | 1 | 2 | 131 |
